# Aircraft noise control policy and mental health: a natural experiment based on the Longitudinal Aging Study Amsterdam (LASA)

**DOI:** 10.1136/jech-2020-214264

**Published:** 2020-11-04

**Authors:** Liming Li, Ludovico Carrino, Erica Reinhard, Erik Timmermans, Martijn Huisman, Jip Claassens, Jeroen Lakerveld, Mauricio Avendano

**Affiliations:** 1 Department of Global Health & Social Medicine, King’s College London, London, UK; 2 Department of Sociology, University of Cambridge, Cambridge, UK; 3 Department of Economics, Ca’ Foscari University of Venice, Venice, Italy; 4 Department of Public Health, Erasmus Medical Center, Rotterdam, Netherlands; 5 Department of Epidemiology and Biostatistics, Amsterdam UMC - Location VU University Medical Centre, Amsterdam Public Health Research Institute, Amsterdam, Netherlands; 6 Department of Sociology, Faculty of Social Sciences, Vrije Universiteit Amsterdam, Amsterdam, Netherlands; 7 Spatial Information Laboratory (SPINlab), Vrije Universiteit Amsterdam, Amsterdam, Netherlands; 8 Department of Epidemiology and Biostatistics, Amsterdam Public Health Research Institute, Amsterdam UMC, Vrije Universiteit Amsterdam, Amsterdam, Netherlands; 9 Upstream Team, Amsterdam UMC, Amsterdam, Netherlands; 10 Julius Centre for Health Sciences and Primary Care, University Medical Centre Utrecht, Utrecht, Netherlands; 11 Department of Social and Behavioral Sciences, Harvard T.H. Chan School of Public Health, Harvard University, Boston, USA

**Keywords:** Mental health, noise, longitudinal studies

## Abstract

**Background:**

This study examines the impact of environmental noise policy on depressive symptoms by exploiting the national experiment afforded by the New Deal aircraft noise control policy introduced in Schiphol (Amsterdam) in 2008.

**Methods:**

Data came from older adults (ages 57–102) participating in three waves (2005/2006, 2008/2009 and 2011/2012) of the Longitudinal Aging Study Amsterdam (LASA) (N=1746). Aircraft noise data from the Netherlands Environmental Assessment Agency were linked to LASA cohort addresses using the GeoDMS software. The Centre for Epidemiologic Studies—Depression (CES-D) scale was used to measure depressive symptoms. Using a difference-in-dfferences (DiD) approach, we compared changes in CES-D levels of depressive symptoms before and after the policy between people living close (≤15 km) and those living far away (>15 km) from Schiphol airport.

**Results:**

There were few changes in noise levels after the introduction of the policy. Estimates suggested that the policy did not lead to a reduction in noise levels in the treatment areas relative to the control areas (DiD estimate=0.916 dB(A), SE=0.345), and it had no significant impact on levels of depressive symptoms (DiD estimate=0.044, SE=0.704). Results were robust to applying different distance thresholds.

**Conclusion:**

The New Deal aircraft noise control policy introduced in Amsterdam was not effective in reducing aircraft noise levels and had no impact on depressive symptoms in older people. Our results raise questions about the effectiveness of the current noise control policy to improve the well-being of residents living near the airport.

## INTRODUCTION

Exposure to aircraft noise has increased rapidly over the last decades in response to rising demand for air transportation. Research suggests that higher levels of aircraft noise are associated with increased risk of chronic diseases such as hypertension and cardiovascular disease, prescription medication rates and mortality.^1–4^ There is less consensus, however, on whether aircraft noise is also associated with mental health. In addition, most studies have focused on the general population, but there is limited evidence of how aircraft noise impacts older people.

Aircraft noise may increase depressive symptoms through a variety of mechanisms. Prior studies suggest that noise induces stress^5 6^ once it exceeds individuals’ perceived resources for coping,^7^ which may in turn lead to depressive symptoms such as fatigue, lack of concentration or reduced enjoyment. Environmental noise also disturbs sleep,^6 8–10^ which has been linked to depression.^11^ Annoyance due to environmental noise may also increase social isolation by limiting outdoor activity and social interactions, thus increasing the risk of mental illness.^9^

Current studies provide limited evidence on the association between aircraft noise and depressive symptoms among older adults, as most studies have focused on the general or younger population.^12^ So far, studies have not found a consistent age-patterning in the effect of noise on overall health. For example, some studies suggest that older people are at lower risk of reporting feeling annoyed and disturbed by noise than middle-aged adults, but they are at increased risk of cardiovascular risk due to noise exposure.^13^ The prevalence of depression in older adults in Europe ranges from 18% to 37%^14^ and is considered a major cause of reduced social function and well-being in older age.^15^ Older people may be at increased risk due to environmental noise due to their lower residential mobility, increased time spent at home and higher sensitivity to environmental influences on sleep disturbance and annoyance.^16^

An innovative approach to study this question comes from the natural experiment afforded by recent noise control policies introduced across major city airports. These policies grew largely in response to European Directive 2002/49/EC, which requires member states to provide noise maps and action plans every 5 years.^17^ Several major airports have introduced noise control measures such as restricting flights in early morning hours and changing flight routes to reduce noise in highly affected areas. In addition, cities have revamped efforts to ensure availability of data on airport noise and control measures, enabling researchers to evaluate their impact.

To our knowledge, very few studies have examined whether European noise control policies have led to changes in mental health outcomes.^4^ We contribute to the literature by exploiting a natural experiment, namely, a noise control policy introduced in Schiphol airport near Amsterdam, the Netherlands. Schiphol is the world’s fifth busiest international airport in terms of traffic. To our knowledge, this is the first study that links longitudinal data of individuals followed for a decade of rising aircraft noise to a specific noise-reduction policy.^18^ We focus on older people and investigate whether current policy efforts influence the mental well-being of people living near city-airports.

## POLICY: THE SCHIPHOL NEW DEAL

The 2002 new Aviation Act reduced the quota of flight movements around Schiphol airport and defined ‘enforcement points’ for noise emission levels. While people living within the ‘enforcement points’ experienced lower noise levels than before, those outside the enforcement area reported higher noise-related annoyance and sleep disturbance.^19^ In response, the ‘Nuisance Limit and Schiphol Development Deal’ was ratified in late 2008, introducing more drastic measures by changing flight routes and extending night procedures. *The Schiphol Deal* aimed to reduce by 5% the number of individuals experiencing disturbance inside the 48 A-weighted decibels (dB(A)) Level day–evening–night (Lden) contour. By 2012, two-thirds of the proposed noise-reduction measures had been implemented.^18^

## METHODS

### Data and measures

#### Longitudinal Aging Study Amsterdam

Longitudinal Aging Study Amsterdam (LASA) is an ongoing longitudinal cohort study in the Netherlands that studies the determinants, trajectories and consequences of physical, cognitive, emotional and social functioning in older adults. The baseline LASA sample of 3107 older adults was drawn from three regions (around Zwolle, Oss and Amsterdam) in 1992/1993.^20 21^ We included data from waves 2005/2006 (N=2165), 2008/2009 (N=1818) and 2011/2012 (N=1523), as older adults in these waves were exposed to aircraft noise before and after the Schiphol New Deal.

We excluded respondents without complete information on distance from Schiphol airport and CES-D depression score, as well as respondents who moved from living close to Schiphol to living far from Schiphol between waves, using a distance of 15 km as the threshold. After dropping respondents with incomplete information in their demographic and socioeconomic characteristics, we had a total sample of 1746 persons with 3431 observations.

#### Depressive symptoms

We measured depressive symptoms with the Centre for Epidemiologic Studies—Depression (CES-D) scale, a validated 20-item measure to detect both clinical and non-clinical depressive symptoms in the general population. CES-D evaluates major components of depressive symptomatology such as depressed mood, feelings of worthlessness, helplessness and hopelessness. Participants in LASA with missing values for five or more items were excluded. The total score ranged from 0 to 60, with higher scores indicating higher levels of depressive symptoms.^22^

#### Aircraft noise exposure

Data on aircraft noise exposure came from the Netherlands Aerospace Centre, commissioned by the Netherlands Environmental Assessment Agency. Daily average cumulative aircraft noise levels from Schiphol airport were modelled in 2005, 2008 and 2012 for raster cells of 25×25 m in the Netherlands. Noise was measured in Lden, expressed in A-weighted decibels (dB(A)) and linked to the point locations of addresses using the GeoDMS software (Object Vision BV, Amsterdam) and the *Addresses and Building key register* of the Netherlands’ Cadastre, and the *Land Registry and Mapping Agency* from 2012.

The address-level aircraft noise data for 2005, 2008 and 2012 were first aggregated at six-digit postcode level and then linked to LASA participants in the 2005/2006, 2008/2009 and 2011/2012 waves, respectively. We used aircraft noise as both a continuous variable and a dichotomous variable using 48 dB(A) to estimate the effects of the policy target on individuals living within and outside this policy-relevant noise contour, which also enabled us to identify non-linearities that would be missed when using the continuous version of the variable. Distance between the centroid of participants’ six-digit postcode area and the centroid of Schiphol Airport’s six-digit postcode was calculated in metres.

In addition, we incorporated information on participants’ age, marital status, employment, retirement, household income and physical functioning. Detailed variable definitions were provided in online supplemental appendix 1.

10.1136/jech-2020-214264.supp1 Supplementary data

### Analytical approach and identification strategy

We used a difference-in-differences (DiD) approach to identify the impact of the *Schiphol New Deal* policy. First, we estimated the impact of the policy on aircraft noise levels, by comparing changes in noise levels before and after the implementation of the policy for individuals in households located close to the airport (treatment group), and compared these to changes in noise levels for individuals living far from the airport (control group). Treatment was defined as living closer than 15 km from Schiphol Airport. For robustness, we also presented results using an alternative threshold of 10 km. The model specification was as follows:

1 $$\begin{array}{l} Noise_{ipt}=\beta_{0}+\beta_{1}time_{t}+\beta_{2}airport\_proximity_{p}+\beta_{3}time_{t}*\\ \;airport\_proximity_{p}+\beta_{4}X_{ipt}+\varepsilon_{ipt} \end{array}$$

where *noise* is for daily average aircraft noise levels from Schiphol Airport in Lden (dB (A)) at six-digit postcode level for individual *i*, living in postcode *p*, interviewed at time *t*; we first used noise as a continuous variable to estimate the extent of changes in noise levels due to the policy intervention. We then included a dichotomous aircraft noise variable (using 48 dB(A) Lden as the threshold) to estimate the effects of the policy target on individuals living within and outside this policy-relevant noise contour; time is a binary variable taking value 1 for the post-policy period (2012) and 0 otherwise; airport_proximity takes value 1 if individuals live within 15 km from the airport and 0 otherwise; and time×airport_proximity corresponds to the differences in change between the treated and control groups. The coefficient of the interaction term $\beta_{3}$ is the double difference computed at the mean value of the outcome.

Second, we compared changes in depression symptoms before and after the implementation of the policy for individuals living close to the airport, and compared these to changes in depression symptoms for individuals living far away from the airport. As before, treatment was defined with a threshold of 15 km distance from Schiphol Airport. Our main specification was as follows:

2 $$\begin{array}{l} mental\;health_{ipt}=\gamma_{0}+\gamma_{1}time_{t}+\gamma_{2}airport\_proximity_{p}+\\ \;\gamma_{3}time_{t}*airport\_proximity_{p}+\gamma_{4}X_{ipt}+\eta_{ipt} \end{array}$$

where mental health refers to CES-D score; time, airport_proximity and time×airport_proximity have the same definition as in equation (1). The coefficient of the interaction term $\gamma_{3}$ is computed at the mean value of the outcome and corresponds to the differences in change between the treated and control groups (the DiD estimate). We also conducted supplementary analyses of the policy impact on life satisfaction, anxiety, sleep, cognition and loneliness, as these variables have been shown to be associated with depressive symptoms^5 8 12 22 23^ and may provide insights into some of the potential mechanisms that might link aircraft noise to depressive symptoms in older age.

A key assumption of the DiD approach is that the control group offers a good counterfactual of what the changes in levels of depressive symptoms would have been in the treatment group, in the absence of the policy.^24^ We tested the common-trend assumption by looking at trends prior to the policy. We reported robust SEs clustered at the postcode level.

Statistical analyses were carried out in RStudio (version 1.1.383), and graphs were done using STATA (version 15.1).

## RESULTS

Table 1 reports descriptive data on our sample. Respondents living closer to the airport were exposed to higher levels of aircraft noise and reported higher average levels of depressive symptoms relative to those living farther away. Both groups were similar in terms of sociodemographic characteristics, except for marital status.

**Table 1:** Descriptive statistics, by treatment and control groups

Variables	Full sample	Control group	Treatment group	Difference (p value)
CES-D score	8.132 (7.178)	7.996 (7.153)	8.771 (7.263)	0.016
Aircraft noise	45.714 (3.106)	44.955 (3.073)	46.699 (2.864)	<0.001
Age	73.141 (8.548)	73.133 (8.512)	73.178 (8.721)	0.908
Married or living with partner	62.5% (0.484)	65.1% (0.477)	50.5% (0.500)	<0.001
Currently in paid employment or is self-employed	13.2% (0.339)	13.0% (0.337)	14.1% (0.349)	0.479
Currently retired	17.5% (0.380)	17.4% (0.379)	17.9% (0.384)	0.748
Net annual household income above the country-specific net mean household income	40.6% (0.491)	41.4% (0.493)	36.9% (0.483)	0.037
Having difficulty walking 2–3 blocks or for about 400–500 m or for 5 min	79.9% (0.401)	80.0% (0.400)	79.4% (0.405)	0.758
N	1746	1426	320	

Mean values are reported. SD are included in brackets.

The sample size for aircraft noise is significantly smaller due to missingness in noise levels (N=727, with 403 in the control group and 324 in the treatment group).

Details of the samples with and without complete noise information can be found in online supplemental appendix 2.

Stars represent statistical significance, as follows: *p<0.05; **p<0.01; ***p<0.001.

CES-D, Centre for Epidemiologic Studies—Depression.

**Figure 1:**
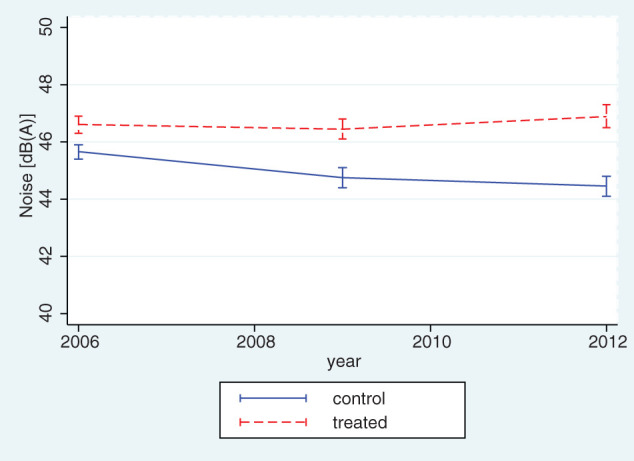
Aircraft noise levels by treatment status (living within 15 km from the airport or not) before and after the policy intervention, 2006–2012, N=727.

**Figure 2:**
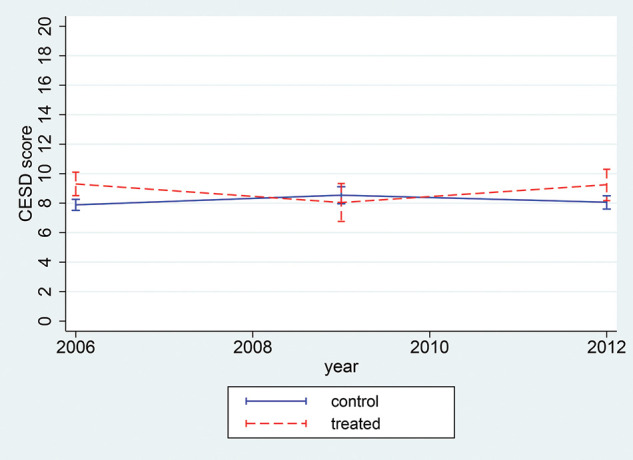
CES-D levels of depressive symptoms by treatment status (living within 15 km from the airport or not) before and after the policy intervention, 2006–2012, N=1746. CES-D, Centre for Epidemiologic Studies—Depression.

Figure 1 shows the average level of aircraft noise exposure separately for treatment and control areas in LASA. Noise levels remained constant in control areas (far from the airport), while they decreased in 2009 before increasing again in 2012 in treatment areas (close to the airport). Figure 2 shows that levels of depressive symptoms remained constant in treated areas, while control areas experienced a decline over the study period, leading to a widening gap between the two groups.

We then investigated the impact of the policy on noise levels by comparing trends between treatment and control areas. The results are shown in table 2. Model 1 in table 2 assigned treatment based on a distance of 15 km to Schiphol. Respondents living close to the airport were exposed to significantly higher levels of noise (β=1.48, SE=0.199). The DiD estimate was positive (β=0.916, SE=0.345), which suggested that the policy did not lead to a reduction in noise levels in the treatment areas relative to the control areas. Results from figure 1 suggested that this was explained by the fact that noise levels did not change in treatment areas, while there was a small decline in control areas. This difference in trends was however relatively small and suggests overall that the policy had very little impact on noise levels.

**Table 2:** Difference-in-difference: OLS models of the impact of the New Deal policy in Schiphol on aircraft noise levels in LASA participants, 2005–2012

	Model 1 (15 km as threshold) β	Model 2 (10 km as threshold) β
1. Policy exposure (year=2012)	−0.053 (0.487)	0.206 (0.434)
2. Treatment group (=close to Schiphol)	1.48*** (0.199)	3.114*** (0.187)
3. Policy exposure×Treatment	0.916* (0.345)	0.768* (0.341)

Robust SEs clustered at the postcode level are reported.

Further covariates include participants’ age, marital status, employment, retirement, household income, physical functioning and year.

Stars represent statistical significance, as follows: *p<0.05; **p<0.01; ***p<0.001.

LASA, Longitudinal Aging Study Amsterdam; OLS, ordinary least squares.

Table 3 shows the results from the DiD models that estimate the association between noise-reduction policies and depressive symptoms. Results from Model 1 (threshold of 15 km) suggested that depressive symptoms increased in the control group between the pre-policy and post-policy period (β=1.279, SE=0.635). However, the DiD estimate suggested that the policy had no statistically different impact on depressive symptoms for the treated group when compared to the control group (β=0.044, SE=0.704). Results of the DiD estimate using the 10 km threshold conveyed a similar message.

**Table 3:** Difference-in-difference: OLS models of the impact of the New Deal policy in Schiphol on CES-D levels of depressive symptoms in LASA participants, 2005–2012

	Model 1 (15 km as threshold) β	Model 2 (10 km as threshold) β
1. Policy exposure (year=2012)	1.279* (0.635)	1.212 (0.630)
2. Treatment group (=close to Schiphol)	0.290 (0.369)	0.762 (0.491)
3. Policy exposure×Treatment	0.044 (0.704)	0.570 (0.971)

Robust SEs clustered at the postcode level are reported.

Further covariates include participants’ age, marital status, employment, retirement, household income, physical functioning and year.

Stars represent statistical significance, as follows: *p<0.05; **p<0.01; ***p<0.001.

CES-D, Centre for Epidemiologic Studies—Depression; LASA, Longitudinal Aging Study Amsterdam; OLS, ordinary least squares.

We also assessed the impacts of the policy on life satisfaction, anxiety, sleep, cognition and loneliness,^5 8 12 22 23^ and we found no evidence that the policy affected these outcomes (online supplemental appendix 3). In sensitivity analyses, we evaluated the effects of the policy on CES-D subcomponents^25^ and CES-D caseness depression (CES-D scores of 16 or higher are considered as predictors of clinical depression).^26^ As before, we found no significant effect of the policy (online supplemental appendix 4). We also experimented with different definitions of the distance thresholds from 10 km to 20 km, and the results consistently showed the policy had no impact on either aircraft noise or CES-D depressive symptoms for LASA participants (online supplemental appendix 5).

Finally, we tested the parallel trend assumption by examining differences in trends in noise and depressive symptoms for treatment and control groups prior to the policy. There were no significant differences in both noise and depression trends between treatment and control groups in the period 2002–2007, using the 15 km distance-threshold for either noise or depression, as well as for depression using the 10 km threshold (online supplemental appendices 6–7). These results yielded support for the common trend assumption.

## DISCUSSION

Our study found no evidence that a policy that aimed to reduce aircraft noise in Schiphol airport reduced noise levels. Unexpectedly, we also found no evidence that the policy reduced depressive symptoms in older people living close to the airport. Our results suggest that existing policies may not be effective in reducing aircraft noise and have therefore a limited potential to impact the mental well-being of older people.

To our knowledge, few studies have examined the impact of aircraft noise control policies on mental health. An exception is a recent study that examined the impact of a 5-month trial, which changed early morning patterns of aircraft landings at London Heathrow airport.^4^ This study found that relative to control regions, areas subject to the trial experienced significant declines in prescribed drugs for respiratory and central nervous system conditions, which included antidepressants and drugs to treat insomnia. However, this study used aggregate prescription data and did not have individual-level data on more comprehensive measures of mental health such as depression or anxiety.

Several studies have found an association between aircraft noise and depressive symptoms. However, some studies suggest that this association may be due to confounding by socioeconomic factors or other demographic characteristics.^27^ In our study, older people living near the airport had on average higher levels of depressive symptoms than those living further away prior to the policy, but this difference was not statistically significant (table 3). Our study, however, does not necessarily question the hypothesis that aircraft noise is unrelated to depressive symptoms, but instead highlights the fact that a major policy that aimed to reduce aircraft noise levels appeared to be ineffective in reducing noise and improving the mental well-being of people residing near the airport.

### Strengths and limitations

Our study differs from earlier studies in two important dimensions. First, we examined the impact of a policy that aimed to reduce noise levels. It is possible that changes in noise levels require longer periods of exposure, and larger magnitude of change, to reduce depressive symptoms. Second, our study focused on older people, whereas prior studies have focused primarily on young adults and adolescents.^9 28–31^ A potential hypothesis is that older people have greater ability to regulate their emotions and view situations positively, and they place more attention on goals from which they derive psychological well-being than younger people.^32 33^ As a result, older people may be less troubled with stress and anger as they age,^34^ and they may be less likely to be affected by environmental exposures such as aircraft noise.

Our study has several limitations. First, due to data availability, our data on aircraft noise are based on postcodes instead of addresses. However, existing literature provides no evidence that aircraft noise exhibits greater variations at the postcode level than at the address level.^2^ Moreover, we use noise data at the level of six-digit postcodes, which constitutes the smallest geographical unit available in the Netherlands with an average of 50×50 m in size and includes 10–20 households and are sufficiently informative for our study. Linking the postcode level noise data to individuals also has the strength of safeguarding the privacy of individuals’ residential addresses.^35^

Second, our results are based on respondents with complete data. To assess the impact of selection, we compared sample characteristics of participants with and without complete information on noise. Results showed that both samples were similar except for marital status. However, a closer examination indicated that these two subsamples did not differ in terms of the policy effects on noise and mental health (results available upon request). These results suggest that sample selection is unlikely to account for our results.

Finally, we select the 15 km threshold as our main specification, because it is a relatively narrow definition of distance from the airport while allowing a reasonable sample size for the treatment group. While a smaller threshold would have been preferable, sensitivity analysis showed that our results were very similar when we used smaller or larger distance thresholds (online supplemental appendix 5).

## CONCLUSION

Our results indicated that tighter noise control measures in Schiphol airport did not reduce noise levels and had no effects on the mental health of older people living close to the airport. Notwithstanding methodological limitations, these findings suggest that existing noise control policies may need to be revised or expanded to generate significant changes. Further research should examine whether more comprehensive policies in other airports may have had larger effects on noise and the mental well-being of older people.

**What is already known on this subject**

- Aircraft noise has been shown to be associated with health among people living in proximity to airports.

**What this study adds**

- A policy to reduce aircraft noise in one of the largest world airports was not effective in reducing noise exposure levels and had no impact on the mental well-being of older residents living near the airport.
