# Supplementary material for: Aircraft noise control policy and mental health: a natural experiment based on the Longitudinal Aging Study Amsterdam (LASA)
Source: J Epidemiol Community Health. 2020 Nov 4;75(5):458–63. doi: 10.1136/jech-2020-214264 (PMC8053324; doi:10.1136/jech-2020-214264)
Supplement: Supplementary data [file jech-2020-214264supp001.pdf]

ONLINE APPENDIX

Appendix 1 Definitions and details of LASA variables

Variable name	Description
Aircraft noise	Aircraft noise was measured in Lden (Level day-evening-night) and expressed in A-weighted decibels [dB(A)]. The measure Lden accounts for the fact that noise in the evening and the night cause more annoyance than noise during the day. The average noise levels during the day (7:00-19:00 hours), the evening (19:00-23:00 hours) and the night (23:00-7:00 hours) were calculated first and the levels of noise in the evening and the night are increased with 5 and 10 dB (A), respectively. Subsequently, the daily mean noise was calculated by dividing the noise levels during day, evening and night by 3. The noise level of a particular raster cell was linked to the point locations of all addresses that fell within that specific raster cell using GeoDMS software. We used levels of noise as both a continuous and a dichotomous variable (48 dB(A) as threshold) in our analysis.
CES-D	In LASA the CES-D 20-item depression scores (0-60) were calculated for participants with data on at least 16 items (out of a possible 20). If 1 or more items were missing, scores on the completed items were summed and divided by the number of items completed, then multiplied by 20. If participants missed 5 or more items, scores were not calculated and coded as missing.
Somatic/vegetative signs	Component of CES-D, measured by the following question: (1) I was bothered by things that usually don't bother me; (2) I did not feel like eating; (3) I had trouble keeping my mind on what I was doing; (4) I felt that everything I did was an effort; (5) My sleep was restless; (6) I could not get going.
Interpersonal distress	Component of CES-D, measured by the following question: (1) People were unfriendly; (2) I felt that people disliked me.
Positive affect (reversely coded)	Component of CES-D, measured by the following question: (1) I felt that I was just as good as other people; (2) I felt hopeful about the Future; (3) I was happy; (4) I enjoyed life.
Depressed affect	Component of CES-D, measured by the following question: (1) I Felt that I could not shake of the blues even with help From my family or friends; (2) I felt depressed; (3) I felt lonely; (4) I had crying spells; (5) I felt sad.
High levels of depressive symptoms	A dichotomous variable that takes value if CES-D score is 16 or higher and value 0 otherwise.

Low life satisfaction	A dichotomous indicator of whether the participant is in the lowest quartile of life satisfaction measured by the scale- or item- specific life satisfaction score.
Anxiety scores	Anxiety symptom score ranging from 0 to 21.
Having elevated anxiety symptoms	A dichotomous indicator of whether the participant has elevated anxiety symptoms in accordance with the HADS-A threshold from Zigmond & Snaith (1983) and Snaith (2003).
Having difficulty falling asleep	A dichotomous indicator of whether the participant sometimes had difficulty falling asleep.
Cognitive scores	Current cognition score ranging from 0 to 30 measured by the Mini Mental State Examination (MMSE).
Having cognitive impairment	A dichotomous indicator of whether the participant has cognitive impairment in accordance with the Mini Mental State Examination (MMSE) threshold from Lezak, Howieson and Loring (2004).
High loneliness	A dichotomous indicator of whether the participant is in the top quintile of loneliness measured by the scale-specific loneliness score.
Age	A continuous variable indicating the age of LASA participants when surveyed
Marital status	A dichotomous variable indicating whether the participants was married or living with partner when surveyed
Employment status	A dichotomous variable indicating whether the participants was currently in paid employment or is self-employed when surveyed
Retirement status	A dichotomous variable indicating whether the participants was currently retired when surveyed
Household income	A dichotomous variable indicating whether net annual household income above the country-specific net mean household income
Physical functioning	A dichotomous variable indicating whether the participant shad difficulty walking 2 to 3 blocks or for about 400 to 500 meters or for 5 minutes when surveyed
Year	A continuous variable indicating the year of the participants being surveyed

Appendix 2 Comparison of sample characteristics

Variables	Overall sample	Sample with missing noise information	Sample with noise information	Difference (P-value)
CES-D score	8.132<br>(7.178)	8.075<br>(7.327)	8.215<br>(6.954)	0.577
Age	73.141<br>(8.548)	72.929<br>(8.438)	73.452<br>(8.700)	0.079
Married or living with partner	0.625<br>(0.484)	0.643<br>(0.479)	0.600<br>(0.490)	0.01
Currently in paid employment or is self-employed	0.132<br>(0.339)	0.132<br>(0.339)	0.132<br>(0.339)	0.983
Currently retired	0.175<br>(0.380)	0.169<br>(0.375)	0.183<br>(0.387)	0.31
SRH being poor	0.406<br>(0.491)	0.402<br>(0.490)	0.413<br>(0.493)	0.494
Having difficulty walking 2 to 3 blocks or for about 400 to 500 meters or for 5 minutes	0.799<br>(0.401)	0.797<br>(0.402)	0.801<br>(0.400)	0.812
N	1,746	1,019	727	

Notes: Stars represent statistical significance: *p < 0.05. **p < 0.01. ***p < 0.001. Mean values are reported. Standard deviations are included in brackets.

Appendix 3 Difference-in-difference: OLS models of the impact of the New Deal policy in Schiphol on other mental health related outcomes in LASA participants, 2005-2012

	Low life satisfaction	Anxiety scores	Having elevated anxiety symptoms	Having difficulty falling asleep	Cognitive scores	Having cognitive impairment	High levels of loneliness
15km as threshold	β	β	β	β	β	β	β
i. Policy exposure (year =2012)	0.022<br>(0.060)	0.588<br>(0.457)	0.037<br>(0.026)	-0.024<br>(0.088)	-0.381<br>(0.374)	0.053<br>(0.039)	0.117*<br>(0.059)
ii. Treatment group (=close to Schiphol)	-0.005<br>(0.026)	0.335<br>(0.195)	0.016<br>(0.011)	-0.031<br>(0.034)	0.251<br>(0.164)	-0.009<br>(0.017)	0.019<br>(0.026)
iii. Policy exposure* Treatment	0.034<br>(0.045)	-0.259<br>(0.351)	-0.016<br>(0.022)	-0.020<br>(0.072)	-0.209<br>(0.256)	0.002<br>(0.024)	0.039<br>(0.047)
10km as threshold							
i. Policy exposure (year =2012)	0.035<br>(0.059)	0.566<br>(0.443)	0.038<br>(0.025)	-0.048<br>(0.085)	-0.447<br>(0.375)	0.061<br>(0.039)	0.126*<br>(0.058)
ii. Treatment group (=close to Schiphol)	0.006<br>(0.031)	0.602*<br>(0.242)	0.023<br>(0.015)	-0.010<br>(0.039)	0.401*<br>(0.179)	-0.016<br>(0.019)	0.032<br>(0.031)
iii. Policy exposure* Treatment	0.0007<br>(0.054)	-0.302<br>(0.415)	-0.029<br>(0.026)	0.052<br>(0.086)	-0.056<br>(0.267)	-0.028<br>(0.025)	0.033<br>(0.058)

Notes: Stars represent statistical significance: *p < 0.05. **p < 0.01. ***p < 0.001. Robust standard errors clustered at the postcode level are reported. Covariates are controlled, including participants' age, marital status, employment, retirement, household income, physical functioning, and year.

Appendix 4 Difference-in-difference: OLS models of the impact of the New Deal policy in Schiphol on CES-D components and CES-D caseness (CES-D score>=16) in LASA participants, 2005-2012

	Model 1 (15km as threshold) β	Model 2 (10km as threshold) β
CES-D components		
Somatic/vegetative signs		
i. Policy exposure (year =2012)	0.384	0.386
	(0.245)	(0.244)
ii. Treatment group (=close to Schiphol)	0.052	0.145
	(0.142)	(0.186)
iii. Policy exposure* Treatment	0.104	0.135
	(0.262)	(0.352)
Interpersonal distress		
i. Policy exposure (year =2012)	0.069	0.061
	(0.050)	(0.049)
ii. Treatment group (=close to Schiphol)	0.117***	0.144**
	(0.035)	(0.048)
iii. Policy exposure* Treatment	-0.069	-0.012
	(0.056)	(0.081)
Positive affect (reversely coded)		
i. Policy exposure (year =2012)	0.408	0.383
	(0.257)	(0.257)
ii. Treatment group (=close to Schiphol)	0.333*	0.532**
	(0.160)	(0.204)
iii. Policy exposure* Treatment	-0.254	0.021**
	(0.279)	(0.007)
Depressed affect		
i. Policy exposure (year =2012)	0.248	0.248
	(0.186)	(0.186)
ii. Treatment group (=close to Schiphol)	-0.217	-0.121
	(0.101)	(0.135)
iii. Policy exposure* Treatment	0.271	0.360
	(0.203)	(0.284)
CES-D caseness		
i. Policy exposure (year =2012)	0.090*	0.084*
	(0.043)	(0.043)
ii. Treatment group (=close to Schiphol)	0.035	0.036
	(0.026)	(0.033)
iii. Policy exposure* Treatment	-0.040	0.004
	(0.045)	(0.058)

Notes: Stars represent statistical significance: *p < 0.05. **p < 0.01. ***p < 0.001. Robust standard errors clustered at the postcode level are reported. Covariates are controlled, including participants' age, marital status, employment, retirement, household income, physical functioning, and year.

Appendix 5 Difference-in-difference: OLS models of the impact of the New Deal policy in Schiphol on noise levels and CES-D levels of depressive symptoms in LASA participants, using multiple distance thresholds (10-20km), 2005-2012

	Aircraft noise	CES-D score
10km as threshold	β	β
i. Policy exposure (year =2012)	0.206 (0.434)	1.212 (0.630)
ii. Treatment group (=close to Schiphol)	3.114 (0.187) ***	0.762 (0.491)
iii. Policy exposure* Treatment	0.768 (0.341) *	0.570 (0.971)
11km as threshold		
i. Policy exposure (year =2012)	0.103 (0.463)	1.276 (0.634) *
ii. Treatment group (=close to Schiphol)	2.575 (0.197) ***	0.645 (0.443)
iii. Policy exposure* Treatment	0.867 (0.353) *	0.076 (0.849)
12km as threshold		
i. Policy exposure (year =2012)	0.050 (0.469)	1.280 (0.634) *
ii. Treatment group (=close to Schiphol)	2.258 (0.198) ***	0.600 (0.422)
iii. Policy exposure* Treatment	0.827 (0.352) *	-0.001 (0.803)
13km as threshold		
i. Policy exposure (year =2012)	-0.034 (0.473)	1.252 (0.634) *
ii. Treatment group (=close to Schiphol)	1.941 (0.202) ***	0.586 (0.410)
iii. Policy exposure* Treatment	0.918 (0.353) **	0.148 (0.790)
14km as threshold		
i. Policy exposure (year =2012)	-0.057 (0.481)	1.266 (0.634) *
ii. Treatment group (=close to Schiphol)	1.628 (0.203) ***	0.480 (0.394)
iii. Policy exposure* Treatment	0.981 (0.351) **	0.075 (0.760)
15km as threshold		
i. Policy exposure (year =2012)	-0.053 (0.487)	1.279 (0.635) *
ii. Treatment group (=close to Schiphol)	1.48 (0.199) ***	0.290 (0.369)
iii. Policy exposure* Treatment	0.916 (0.345) *	0.044 (0.704)
16km as threshold		
i. Policy exposure (year =2012)	-0.141 (0.488)	1.266 (0.636) *
ii. Treatment group (=close to Schiphol)	1.647 (0.200) ***	0.264 (0.354)
iii. Policy exposure* Treatment	0.905 (0.338) **	0.105 (0.666)
17km as threshold		
i. Policy exposure (year =2012)	-0.108 (0.495)	1.234 (0.637)
ii. Treatment group (=close to Schiphol)	1.577 (0.202) ***	0.267 (0.345)
iii. Policy exposure* Treatment	0.906 (0.340) **	0.271 (0.655)
18km as threshold		
i. Policy exposure (year =2012)	-0.111 (0.496)	1.249 (0.637) *
ii. Treatment group (=close to Schiphol)	1.561 (0.203) ***	0.267 (0.342)
iii. Policy exposure* Treatment	0.909 (0.341) **	0.192 (0.650)
19km as threshold		
i. Policy exposure (year =2012)	-0.112 (0.497)	1.255 (0.637) *
ii. Treatment group (=close to Schiphol)	1.564 (0.203) ***	0.244 (0.342)
iii. Policy exposure* Treatment	0.906 (0.341) **	0.167 (0.650)
20km as threshold		
i. Policy exposure (year =2012)	-0.088 (0.496)	1.252 (0.637) *
ii. Treatment group (=close to Schiphol)	1.629 (0.204) ***	0.189 (0.340)

iii. Policy exposure* Treatment	0.846 (0.341) *	0.196 (0.647)

Notes: Stars represent statistical significance: *p < 0.05. **p < 0.01. ***p < 0.001. Robust standard errors clustered at the postcode level are reported. Covariates are controlled, including participants' age, marital status, employment, retirement, household income, physical functioning, and year. At the 10km distance threshold, the number of people in the treatment and control groups are 174 vs 559 for aircraft noise and 175 vs 1,574 for CES-D score outcomes. At the 20km distance threshold, the number of people in the treatment and control groups are 406 vs 327 for aircraft noise and 396 vs 1,356 for CES-D score outcomes.

Appendix 6 Common trend assumption test for aircraft noise: trends in aircraft noise in the period 2002-2009

	Model 1 (15km as threshold) β	Model 2 (10km as threshold) β
Year (Refence=2002)		
2006	0.815*	0.930***
	(0.347)	(0.271)
2009	-0.125	0.125
	(0.347)	(0.276)
Treatment group (=close to Schiphol)	1.538***	4.937***
	(0.441)	(0.456)
Year*Treatment group		
2006* Treatment group	-0.774	-2.053***
	(0.542)	(0.555)
2009* Treatment group	-0.217	-1.411*
	(0.564)	(0.595)

Notes: Stars represent statistical significance: *p < 0.05. **p < 0.01. ***p < 0.001. Robust standard errors clustered at the postcode level are reported.

Appendix 7 Common trend assumption test for CES-D levels of depressive symptoms: trends in CES-D scores 2006-2009

	Model 1 (15km as threshold) β	Model 2 (10km as threshold) β
Year (Refence=2006)		
2009	-0.309	-0.444
	(0.275)	(0.260)
Treatment group (=close to Schiphol)	1.492***	1.729**
	(0.449)	(0.606)
2009* Treatment group	-1.181	-0.819
	(0.643)	(0.861)

Notes: Stars represent statistical significance: *p < 0.05. **p < 0.01. ***p < 0.001. Robust standard errors clustered at the postcode level are reported. Due to large number of missing values for CES-D levels of depressive symptoms in LASA 2001/02, we were unable to carry out tests for common trend using data from this wave.
